# The nuclear receptor 4A family members: mediators in human disease and autophagy

**DOI:** 10.1186/s11658-020-00241-w

**Published:** 2020-11-03

**Authors:** Liqun Chen, Fengtian Fan, Lingjuan Wu, Yiyi Zhao

**Affiliations:** 1 College of Biological Science and Engineering, Fuzhou University, Fuzhou, 350108 China; 2 Institute of Apply Genomics, Fuzhou University, Fuzhou, 350108 China

**Keywords:** NR4A family, Autophagy, Human disease, Nur77, Nurr1, Nor1

## Abstract

The Nuclear receptor 4A (NR4A) subfamily, which belongs to the nuclear receptor (NR) superfamily, has three members: NR4A1 (Nur77), NR4A2 (Nurr1) and NR4A3 (Nor1). They are gene regulators with broad involvement in various signaling pathways and human disease responses, including autophagy. Here, we provide a concise overview of the current understanding of the role of the NR4A subfamily members in human diseases and review the research into their regulation of cell autophagy. A deeper understanding of these mechanisms has potential to improve drug development processes and disease therapy.

## Introduction

Autophagy is an evolutionarily conserved catabolic process that attenuates cellular stress by digesting cytoplasmic contents and disposing of intracellular waste [1, 2]. The process involves lysosomal enzymes degrading pathogens, longevity proteins, damaged organelles and other components [3, 4]. Rapid adaptation to environmental changes to maintain homeostasis is key to the health and survival of cells and organisms. By maintaining a balance between synthesis and degradation, autophagy can regulate homeostasis [5].

The process of regulating autophagy is highly conserved, being found in all eukaryote cells [6, 7]. Most cells and tissues have basic autophagy activity under normal physiological conditions. A variety of stimuli can also induce autophagy: for example, cellular stress factors, including physiological stress (e.g., nutritional deficiencies, high temperature, high-density conditions and hypoxia), hormones (e.g., glucagon), and pharmacological agents (e.g., Torin 1 and rapamycin) [6]. Dysregulated autophagy is associated with infections, cancers, and neurodegenerative, metabolic, cardiovascular and lung diseases, among other conditions [3, 8].

The main steps of autophagy are: (1) induction of cell autophagy; (2) nucleation of autophagosomes; (3) amplification and completion of autophagosomes; (4) docking and fusion of autophagosomes and vacuoles; and (5) degradation and outflow of decomposition products [9].

**Fig. 1:**
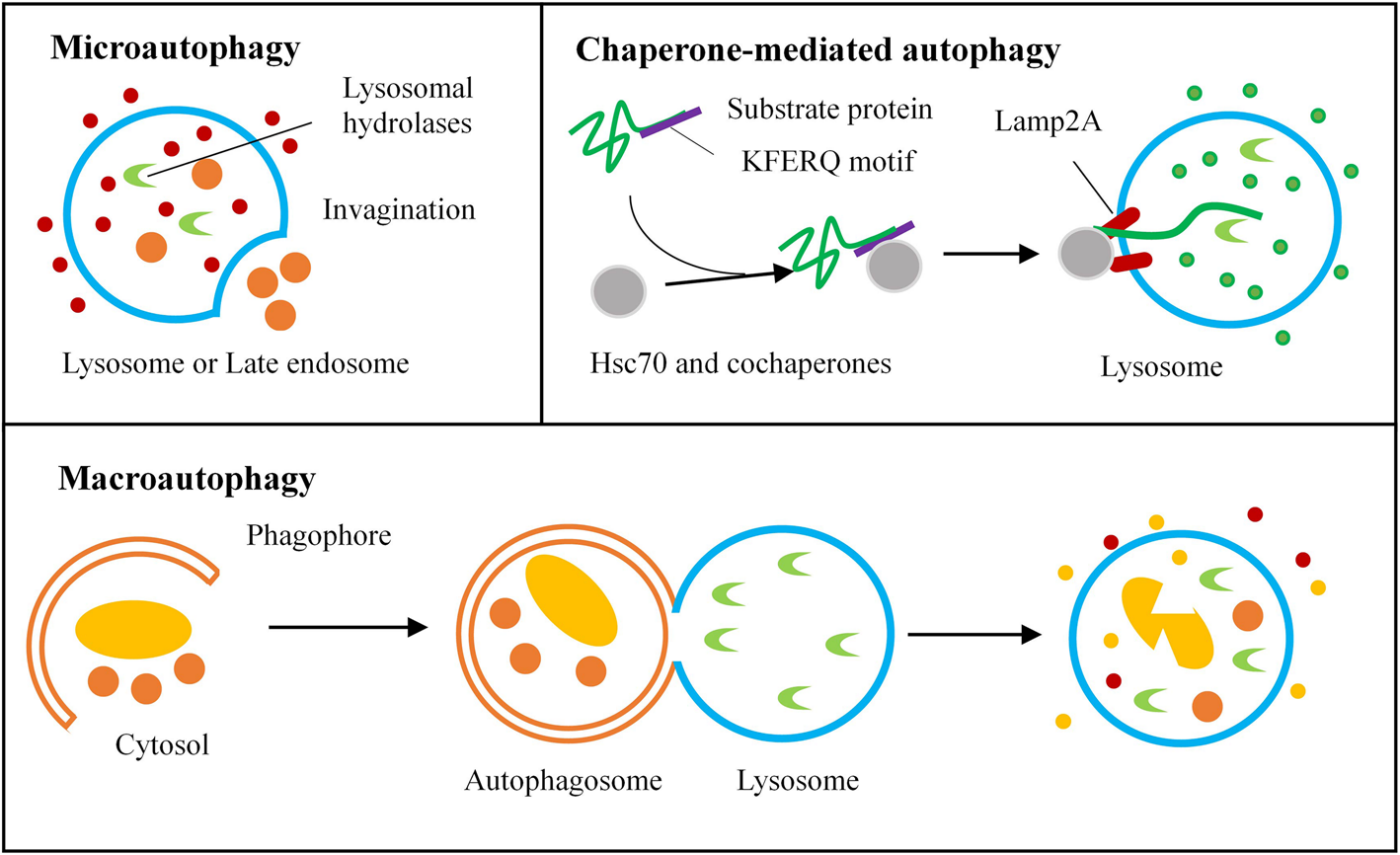
The three main types of autophagy in mammalian cells. Microautophagy: small pieces of the cytoplasm are directly engulfed by lysosomal or late endosomal membranes. Chaperone-mediated autophagy (CMA): unfolded proteins are directly transported across the lysosomal membrane. Macroautophagy: Cytosolic double-membrane autophagosomes form to sequester and transport cargo to the lysosome

In mammals, there are three major types of autophagy: microautophagy, chaperone-mediated autophagy (CMA) and macroautophagy (Fig. 1) [10]. They differ in the involvement of lysosomes and in terms of the cytosolic targets to be degraded.

Microautophagy is a non-selective lysosomal degradation process that involves the invagination of lysosomal or late endosomal membranes, leading to direct phagocytosis of cytoplasmic components [11].

CMA transports unfolded proteins directly across the lysosomal membrane. Substrate proteins containing a KFERQ sequence are first recognized by heat shock cognate protein 70 (hsc-70) and co-chaperones [12]. They are then translocated into the lysosome after binding to lysosomal-associated membrane protein 2A (Lamp-2A) [13].

Macroautophagy is the most important type for maintaining homeostasis because it regulates the turnover and function of key organelles [14, 15]. It is a catabolic process in which intracellular components are encapsulated in double-membrane vesicles known as autophagosomes. These eventually fuse with lysosomal late endosomes, where their contents are degraded and recycled into the cytosol [16].

Nuclear receptors (NRs) were first identified as transcription regulators and then found to respond to some ligands and hormones [17]. The NR superfamily comprises 29 subfamilies consisting of more than 200 functional proteins encoded by 48 genes [18]. They participate in the regulation of almost all physiological processes, including growth, development, metabolism, immunity, differentiation and death [19]. The receptor selectively binds to its corresponding ligands or small-molecule compounds, initiates the transcriptional activity of predetermined specific nuclear genes, or exerts its other biological effects [19]. There is considerable evidence that NRs can work via non-genomic actions [20]. They modulate the activity of ion channels, kinases, phosphatases and other enzymes in various types of cells, and act as ligand-activated cytosolic and membrane receptors [21].

**Fig. 2:**
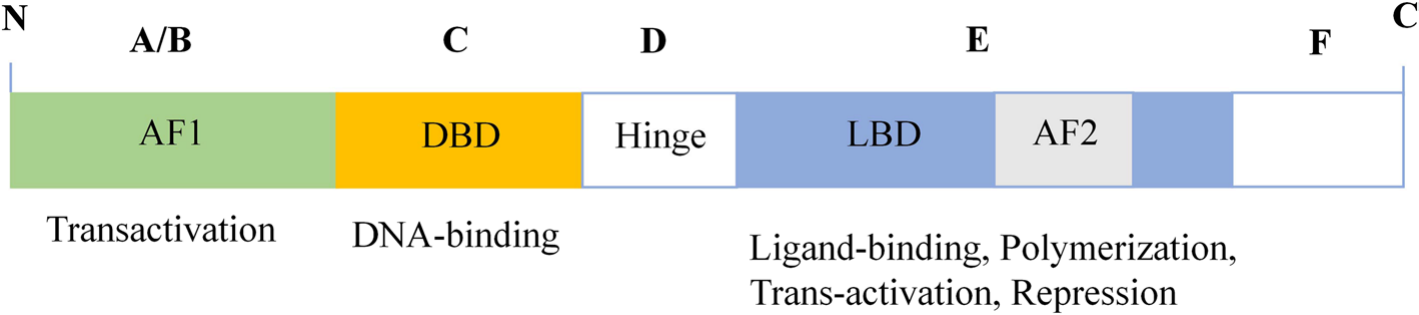
Structural organization of NRs. Most NRs consist of several domains: an N-terminal region with AF1, which is important for its interaction with co-regulators and other transcription factors; a highly conserved zinc-finger DBD, which allows the receptor to bind specific response elements; a variable hinge domain; a ligand-binding domain (LBD) with AF2; and a highly variable C-terminal domain

NRs are usually composed of several domains, with A through F regions from the N-terminus to the C-terminus (Fig. 2) [22, 23]. The length, sequence and structure of the A and B region are highly variable. It contains a transcription activation region, called activation function-1 (AF1), which can activate target genes through interaction with basal transcription factors, co-activators or other transcription factors [24]. The C region is a highly conserved zinc finger DNA-binding domain (DBD) that allows the receptor to bind specific response elements. The D region is a variable hinge region that allows NR proteins to twist or change conformation and usually contains a nuclear localization signal. The E region contains the ligand-binding domain (LBD) of the NR, which includes a ligand-dependent AF2 and consists of 12α-helixes [25]. This region also binds to heat shock proteins and can contain a polymerization region, nuclear localization sequences, a transactivation domain, a transrepression domain and the NRs’ intermolecular block. The AF2 domain undergoes a conformational change after agonist or antagonist binding to the ligand-binding pocket of the NRs [26]. The binding and conformational change provides the site for co-regulators, leading to the recruitment of transcription factors that initiate downstream transcription [27]. Some NRs also contain a variable F region at the C-terminus, but no specific function for this region has been found [28].

The NR superfamily is divided into seven subfamilies. The NR0 subfamily contains nuclear receptor subfamily 0 group B member 1 (NR0B1/DAX1) and nuclear receptor subfamily 0 group B member 2 (NR0B2/SHP). The NR1 subfamily contains thyroid hormone receptor, retinoic acid receptor, vitamin D3 receptor, all peroxisome proliferator-activated receptors (PPARs) and retinoic acid receptor-related orphan receptors, Rev-erb receptor, constitutive androstane receptor, pregnane X receptor, and liver X receptor (LXR). The NR2 subfamily consists of the retinoid X receptor members and hepatocyte nuclear factor 4 receptor. The NR3 subfamily contains the sexual and adrenal steroids, such as glucocorticoid receptor, mineralocorticoid receptor, progestin receptor, androgen receptor (AR) and estrogen-related receptors. The NR4 subfamily consists of nerve growth factor-induced gene B (NGFI-B/Nur77), Nur-related protein1 (Nurr1) and neuron-derived orphan receptor1 (Nor1). The NR5 subfamily contains steroidogenic factor-like receptors, such as steroidogenic factor-1 and liver receptor homolog-1. The sole member of the NR6 subfamily is germ cell nuclear factor-1, which does not fit the common criteria of the other groups [29, 30].

A link between NRs and cellular autophagy has been demonstrated. NRs can reduce or aggravate cell autophagy in various physiological and pathological processes, thereby maintaining stable homeostasis of the internal environment. This review summarizes the state of knowledge on the NR4A subfamily members, which are involved in cell autophagy and have received considerable attention in recent years.

## The NR4A subfamily and human disease

The members of the NR4A subfamily are considered orphan NRs. While they have many structural domains that are similar to those of other NRs, their endogenous ligands have not yet been found. The three members are: Nur77 (also known as Nak-1, N10, or TR3), Nurr1 (also known as Not, Rnr-1 or Tinur) and Nor1 (also known as Chn, Minor or Tec) [31–33]. They have 90–95% homology in their DBDs but divergent N-terminal transactivation domains [34]. Their key roles, which include proliferation, apoptosis, DNA repair, cellular stress, memory, endocrinology, neuronal signaling, and hematopoietic, immune and metabolic processes, have been demonstrated in many cell types. Moreover, these receptors are involved in the onset and progression of numerous diseases, such as obesity, atherosclerosis, inflammation and cancer [35].

### NR4A members in metabolic disease

The members of the NR4A subfamily have important roles in metabolic regulation and energy balance in the body. For example, as hormone- and ligand-dependent DNA-binding proteins, they convert endocrine, metabolic and pathophysiological signals into changes in gene expression and the control of metabolic homeostasis [36]. Studies have shown that the expressions of NR4A subfamily members are highly upregulated in the tissues of diabetic individuals [37].

Nur77 stimulates hepatic gluconeogenesis by upregulating several enzyme-encoding genes that catalyze specific biochemical steps [38]. The elevated expression of Nur77 induced by the β-cell transcription factor Nkx6.1 promotes islet β-cell proliferation and plays an important role in the functional restoration of these cells [39]. Nur77 is also involved in lipid metabolism. Its abnormal expression in the liver is implicated in numerous pathophysiological processes, including fat metabolism, cholesterol metabolism, and hepatic steatosis [40].

Nurr1 regulates the expression of multiple genes related to metabolism and gluconeogenesis, suggesting that Nurr1 may also be an important regulator of metabolic diseases [41].

A pathway that affects insulin signaling has been linked to Nor1 expression in microcells to stimulate the replacement of insulin for glucose [42]. Nor1 also plays an important role in fatty acid oxidation [43]. These studies indicate that NR4A exerts a regulatory role in regulating the metabolism.

### NR4A members in inflammation

Inflammation is essential for protection following biological, chemical or physical stimuli, but inappropriate inflammation, which occurs in inflammatory diseases, causes tissue damage. The members of the NR4A subfamily control growth factor and cytokine signaling pathways and play roles in regulating the inflammatory response [44]. They are abnormally expressed in inflamed human synovial tissue and in the epithelial tissues of individuals with psoriasis [45–47].

Nurr1 appears to be associated with inflammatory arthritis. Its expression is higher in synovial tissue and primary synovial cells according to quantitative PCR measurements [48, 49]. Tumor necrosis factor alpha (TNF-α) selectively activates Nurr1 expression in synoviocytes, leading to increased proliferation. Reducing Nurr1 expression using Nurr1 small interfering RNA (siRNA) can reduce synoviocyte proliferation and migration [50]. These findings indicate that Nurr1 positively mediates TNF-α signaling in synovial tissue, which contributes to the activation of synovial cells and increases proliferation and migration. This leads to cartilage and joint inflammation.

Other pro-inflammatory cytokines, such as interleukin-1β (IL-1β), nuclear factor kappa-B (NF-κB) and vascular endothelial growth factor (VEGF), can also activate Nurr1 expression [51]. Nurr1 has inducible and constitutive anti-inflammatory activity in immune cells with monocyte and macrophage lineages and in glial cells, including astrocytes and microglia [47, 52]. In glial cells, the anti-inflammatory effect of Nurr1 is mediated by docking with NF-κB-p65 on the target of the inflammatory gene promoter [52].

Similarly to the other NR4A receptors, Nor1 is upregulated under inflammatory conditions and induced by stressors. It plays an integral role in T-cell receptor-induced apoptosis [53]. Nor1 is also a causative factor in atherosclerosis with inflammatory signals known to stimulate upregulation of nuclear factor kappa B-mediated Nor1 in atherosclerotic cells [54].

### NR4A members in cancer

The activity of the NR4A subfamily members in cancer is regulated by various cellular signaling pathways, including the protein kinase, Wnt and mitogen-activated protein kinase (MAPK) pathways.

Nur77 is overexpressed in colon, lung and breast cancers (both ER-positive and ER-negative types) [55]. Nurr1 expression is significantly higher in human prostate cancer tissue than in normal prostate tissue [56]. In colorectal cancer cells, ectopic expression of Nurr1 may increase the response to 5-FU and oxaliplatin [57]. Nor1 is overexpressed in breast tumor tissues, with a recent report showing that its expression is higher in triple-negative breast cancer [58]. The combined loss of Nor1 and Nur77 in mice leads to acute myeloid leukemia, demonstrating their role in this disease [59].

Defects in the MAPK pathway are observed in most melanomas, accompanied by an increase in oncogenic BRAF [60]. In melanoma, such defects cause dysregulation of Nur77 and Nurr1 expression, which may be a contributing factor in tumorigenesis. The tumor-specific functions and development of new ligands of NR4A members should be further investigated.

### NR4A members in neurological disease

The NR4A family is closely related to nerve and neurological diseases. Nur77 is expressed in various regions in the brain. After in vivo treatment with 1-methyl-4-phenyl-1,2,3,6-tetrahydropyridine (MPTP), Nur77 expression in the nigrostriatal region reduced significantly [61, 62]. MPTP-induced dopaminergic neuron loss is more severe in Nur77^−/−^ mice than in wild-type mice [63]. In a non-human primate model of Parkinson’s disease, dopamine denervation and chronic l-dopa treatment regulate its expression, suggesting that strong regulation of Nur77 may be related to the reduction in l-dopa-induced dyskinesia [61]. It is also associated with the response to l-dopa, synaptic remodeling and behavioral changes. It acts as a protective agent for nerve cells, as shown with a model of neural ischemia in vitro [64]. Overexpression of Nur77 significantly improves oxygen and glucose deprivation-induced neural damage, while its knockdown exacerbates these conditions.

Nurr1 is essential for the regulation of multiple physiological functions of the human central nervous system. It is related to memory and learning, with wide expression throughout the brain and in peripheral structures, such as the cortex and hippocampus [47]. Its expression is rapidly induced after stress and damage to the central nervous system. Nurr1 knockout mice are unable to produce midbrain dopaminergic neurons and die shortly after birth [65]. The maintenance and maturation of dopamine neurons in the adult midbrain also require Nurr1, which has a role to play in dopamine synthesis and metabolism. It also regulates the expression of α-synuclein, which is the main protein component of the aggregation of Lewy bodies in Parkinson’s disease [66]. Downregulation of Nurr1 expression increases the expression of α-synuclein and transcription factors.

The importance of Nurr1 in Parkinson’s disease was also emphasized in a recent study that used a synthetic Nurr1 small molecule activator. 1,1-bis(3′-indolyl)-1-(*p*-substituted phenyl) methane (C-DIM) is a protective agent that prevents MPTP-induced dopaminergic loss in neurons and increases Nurr1 expression and nuclear localization in the substantia nigra in Parkinson’s disease mouse models [67].

Nor1 is a cAMP response element-binding protein-regulated gene that plays a role as a histone deacetylase inhibitor to enhance memory. In addition, polymorphisms in the Nor1 gene are related to nicotine addiction in patients with mental illness. Other NR4A gene polymorphisms may also correlate with other receptor-mediated health problems.

## Autophagy regulation by NR4A family members in human disease

### Nur77 and autophagy

Nur77 is located on the human chromosome 12q13.13. Human Nur77 cDNA encodes a polypeptide of 598 amino acids. The Nur77 gene is conserved in zebrafish, frog, dog cow, mouse, rat, macaque and chimpanzee. 160 organisms have orthologs with the human gene Nur77.

Nur77 induces apoptosis by targeting the mitochondria [68, 69]. Celastrol, an effective anti-inflammatory pentacyclic triterpene [70], promotes the translocation of Nur77 from the nucleus to inflamed mitochondria and interacts with tumor necrosis factor receptor-associated factor 2 (TRAF2). This interaction is regulated by an LxxLL motif in TRAF2 and results in inhibition of TRAF2 ubiquitination and induction of Lys63-linked Nur77 ubiquitination [71]. There is a strong and specific interaction between Nur77 and p62/SQSTM1. The ubiquitinated Nur77 resides in the mitochondria, rendering them sensitive to autophagy.

The removal of damaged mitochondria through autophagy (mitophagy) is considered a reasonable mechanism for controlling mitochondrial mass and an effective method for reversing the pathological state of chronic inflammatory diseases. Therefore, mitochondrial targeting of Nur77 and the Nur77-TRAF2 and Nur77-p62/SQSTM1 interactions lead to the clearance of damaged mitochondria and reduce inflammation [72–74].

Two miRNAs from related clusters, miR-34 and miR-449 are strongly correlated with respiratory diseases and are potential biomarkers and therapeutic targets for hypertension [75]. Autophagy also plays a key role in airway inflammation and fibrosis-mediated airway remodeling. In both conditions, the expressions of miR-34 and miR-449 are downregulated, whereas the expressions of the autophagy-related proteins are upregulated. The levels of pro-inflammatory factors and fibrosis-related proteins are significantly higher in surgical patients than in healthy individuals. In BEAS-2B normal bronchial epithelial cells, miR-34 and miR-449 overexpression activates AKT, inhibits insulin-like growth factor-binding protein 3 expression, promotes the nuclear import of Nur77, and downregulates fibrosis-related factors and pro-inflammatory cytokines, thus reducing autophagy. In a mouse model of ovalbumin-induced mouse airway inflammation, airway Nur77 deficiency led to mucus cell proliferation and increased airway damage, indicating that Nur77 is resistant to protection against airway inflammation [76]. Drug-induced melanoma usually leads to autophagic cell death with the participation of Nur77 [77]. In a cell model of Parkinson’s disease, the Nur77 agonist cytosporone B (Csn-B) was shown to promote the degradation of α-synuclein [78]. Its mechanism may be related to increasing autophagy levels.

In PC12 cells lesioned by 6-hydroxydopamine (6-OHDA), Nur77 plays an important role in neuromuscular cytolysis and dopaminergic neurodegeneration [79]. When treated with 6-OHDA, Nur77 translocates from the nucleus to the cytoplasm and endoplasmic reticulum. In addition, co-localization of Tom20 with Nur77 and protein disulfide isomerase with Nur77 are induced. Nur77 activation can significantly increase the efficiency of autophagy, not only through upregulation of Beclin-1/LC-3 and downregulation of p62 but also through upregulation of PTEN-induced kinase 1 and downregulation of Parkin. Nur77 partially exacerbates PC12 cell death by exacerbating mitochondrial damage, promoting ER stress and enhancing autophagy. Thus, Nur77 can be a critical target in Parkinson’s disease therapy [80].

A derivative of Csn-B, 1-(3,4,5-trihydroxyphenyl)nonan-1-one (THPN), triggers human melanoma Mel-11, ME4405 and MM200 cells but does not affect non-melanoma. THPN-induced autophagy has been demonstrated [38]. Direct interaction between THPN and Nur77-LBD helps form a suitable surface that can bind to Nix. Then, Nur77 enters the mitochondrial inner membrane through the translocation enzyme of the outer mitochondrial membrane channel [77]. Similarly to other members of the Bcl-2 family, Bcl-B prevents autophagy by binding and inhibiting Beclin-1. Therefore, autophagy is induced when Bcl-B is occupied by excess Nur77, which is achieved through overexpression of the cytoplasmic DBD construct [81].

Nur77 promotes cigarette smoke-induced autophagic cell death by accelerating the dissociation of Bcl-2 and Beclin-1 in chronic obstructive pulmonary disease (COPD). This condition is characterized by rapid and sustained destruction of alveolar septa, leading to a partially reversible limitation of the airflow. Autophagy is involved in the pathogenesis of cigarette smoke-induced COPD. Cigarette smoke promotes Nur77 expression and nuclear export in vitro and in vivo, thereby increasing cigarette smoke extract-induced autophagy. A549 lung cancer cells, human bronchial epithelial cells, and lung tissues exposed to cigarette smoke or cigarette smoke extract express lower levels of light chain 3 (LC3) and Beclin-1 after knockdown of Nur77 with siRNA-Nur77. Co-immunoprecipitation assays show that cigarette smoke extract promotes autophagy partially through stimulating the interaction between Nur77 and Bcl2, resulting in increased dissociation of Bcl-2 from Beclin-1. By contrast, leptomycin B inhibits the dissociation of Bcl-2 from Beclin-1. Thus, nuclear export of Nur77 is required for the cigarette smoke extract-induced autophagic death of lung cells [82].

Dendrogenin A (DDA) is a natural steroidal alkaloid compound that functions as a ligand for LXR. It can induce Nur77 and Nor1 expression in melanoma cells and cause cell death, triggering excessive autophagy both in vitro and in vivo [83].

Nur77 is highly expressed in cancer cells. Its knockdown by siTR3 can reduce cell growth and induce apoptosis [84]. The activity of Nur77 occurs through the formation of a p300/Nur77/Sp1 complex at the proximal GC-rich surviving promoter [85]. In p53 wild-type cells, siTR3 inhibits the mTORC1 pathway due to the activation of p53 and induction of the p53-responsive gene sestrin 2, which subsequently activates the mTORC1 inhibitor AMPKα [86].

### Nurr1 and autophagy

The human Nurr1 gene is located on chromosome 2q22-q23 and encodes a polypeptide of 598 amino acids that displays a high similarity (99%) with its bovine, porcine, and mouse equivalents [87].

Hydroxychloroquine, a hydroxyl derivative of chloroquine, is an antimalarial and antirheumatic drug [88]. A study on a rat rotenone model found that it has the potential to prevent Parkinson’s disease through Nurr1 modulation, anti-inflammatory effects and autophagy inhibition [88]. Hydroxychloroquine effectively enhanced the expression of Nurr1, exhibited anti-inflammatory effects, which can be confirmed by inhibiting certain pro-inflammatory cytokines, and reduced the activity of glycogen synthase kinase 3 beta (GSK-3β). The findings showed that it overcame certain deleterious effects precipitating neurological damage by inhibiting autophagy and enhancing apoptosis [88].

Similarly, cilostazol mediates Nurr1 and enhanced autophagy, thereby enhancing neuroprotective activity in rat rotenone Parkinson’s disease models. Cilostazol is a phosphodiesterase 3 inhibitor that was recently shown to have good neuroprotective activity in a variety of devastating central nervous system diseases. It can successfully upregulate Nurr1 expression in Parkinson’s disease rats, thereby maintaining the function and integrity of dopaminergic neurons. In addition, it shows anti-inflammatory activity, which is reflected in its inhibition of the global controller of the inflammatory signaling pathway, i.e., the NF-κB signaling pathway, together with its downstream pro-inflammatory cytokines TNF-α and IL-1β, via Nurr1 upregulation and GSK-3β inhibition. Cilostazol is a promising candidate for Parkinson’s disease therapy through regulation of Nurr1 expression and cross-regulation of SIRT-1-induced autophagy and GSK-3β-induced apoptosis [89].

Heat stress and related restrictions of intestinal blood flow can disrupt intestinal integrity. Heat stress reduces the mRNA and protein expression of Nurr1, ZO-1, closure proteins, E-cadherin and claudin-6; increases the mRNA expression of NF-κB and IL-1β; and also increases the protein expression of cleaved caspase-3, facilitating the autophagy process [90].

Cystatin C (CYSC) is a secreted cysteine inhibitor encoded by the Cst3 gene. It is commonly used as a biomarker of renal function and is a potential therapeutic medium against Parkinson’s disease through its involvement in VEGF-induced angiogenesis and enhancement of neuron autophagy in neurovascular units [91]. In vivo studies have demonstrated that when A53T SNCA mice are treated with CYSC, VEGF expression increases and effectively induces the expression of Nurr1 in endothelial cells. During this process, the expression level of the autophagy marker LC3-II is increased and caspase-3 is cleaved [91]. The specific mechanism of action is as follows: VEGF transfers Nurr1 from the cytoplasm to the nucleus by regulating the p-PKC-α/p-ERK1/2-Nurr1 signal, thereby greatly reducing PC12 cell degeneration. Incubation with 6-OHDA significantly reduces the density of Nurr1 protein in the cytoplasm and nucleus, while overexpression of VEGF significantly restores the density of the cytoplasmic and nuclear Nurr1 to normal control levels.

Nurr1 also participates in H_2_O_2_-induced cardiac stem cell apoptosis by promoting autophagy. H_2_O_2_ can induce an increase in its expression after cardiomyocyte injury in cancer stem cells (CSCs). Nurr1 siRNA attenuates H_2_O_2_-induced CSC autophagy and apoptosis, indicating that Nurr1 plays an indispensable role in the survival of CSCs under ischemic conditions. The study also demonstrated that NF-κB and reactive oxygen species (ROS) are involved in H_2_O_2_-induced apoptosis and autophagy, and they were the upstream factors regulating Nurr1. Thus, H_2_O_2_ induces autophagy-dependent apoptosis through the ROS/NF-κB/Nurr1 signaling pathway [92].

### Nor1 and autophagy

Nor1, which was first identified in nasopharyngeal carcinoma cells, has high similarity to the classical nitroreductase of *Salmonella typhimurium* [93]. Human Nor1 is also called human organic solute carrier protein 1 (hOSCP1) and is known to mediate various organic solutes on the placental basement membrane in a pH- and sodium-dependent manner [94]. Nor1 is evolutionarily conserved and its expression is restricted to the brain, respiratory epithelial cells and testes.

Nor1 inhibits the growth of cancer cells by interfering with the metabolism of tumor cells. It also promotes the apoptosis of tumor cells under oxidative stress and hypoxia by inhibiting stress-induced autophagy. In addition, it inhibits epithelial-mesenchymal transition as well as invasion and metastasis of cancer cells by activating the FOXA1/HDAC2-Slug regulatory network [93].

Some scholars have studied the expression and role of Nor1 in adaptive remodeling. One study demonstrated that its expression can be induced by exercise and that it depends on calcium or calcineurin signaling in vitro and in vivo [95]. Analysis of fatigue-resistant transgenic mice expressing Nor1 in their skeletal muscles showed increased hypertrophy and vascularization of muscle tissue. The response of Nor1 to an acute episode of moderate intensity exercise (40 min) depends on calcium- or calcineurin-modulated neural phosphate signals. The endurance phenotypes of these fatigue-resistant transgenic mice are associated with hypertrophy, vascularization of the muscle tissue and autophagy, with Nor1 expression specifically related to cellular autophagy [95]. Compared with their wild-type littermates, the transgenic mice showed higher LC3-II, autophagy protein 5 (ATG5) and autophagy protein 12 (ATG12) in their quadriceps femoris muscle extracts. Furthermore, Nor-1 expression significantly increased autophagosome formation in this model [95].

DDA, a newly discovered cholesterol metabolite, was found to increase the expression of Nur77, Nor1 and LC3 by stimulating transcription of NR4A1 and NR4A3, leading to the formation of autolysosomes [96]. DDA-induced formation of LC3-II is also suppressed by single or double knockdown of Nor1 and Nur77, indicating that the two receptors participate in and mediate DDA-induced lethal autophagy in melanoma cells [96].

Nor1 is also involved in regulating autophagy, metabolism and apoptosis in nasopharyngeal carcinoma. Acute oxidative stress induces Nor1 expression in normal cells and cancer cells. In cancer cells expressing Nor1, autophagy is reduced after oxidative stress is induced, mitochondrial respiration and energy metabolism are inhibited, and apoptosis is enhanced [97]. By contrast, knocking down Nor1 using Nor1 siRNA results in increased autophagy and reduces H_2_O_2_-induced HeLa cell death. These studies show that Nor1 is a regulator of autophagy and reveals a new aspect of the interaction between apoptosis and autophagy.

## Summary and future perspectives

NRs have a huge impact on almost all physiological processes in mammalian cells, tissues and organs, and at all levels of the organism. They serve as an interface between changes in the environment of the cell or system and the genome, facilitating the convergence of a variety of extracellular and intracellular signals and playing an important role in initiating intercellular physiological transcriptional programs. Their close relationship to many human diseases means they have potential as therapeutic targets, including in precision therapy. Advances in chemistry and molecular biology over the past 30 years have provided a greater understanding of the number, regulation and function of NRs, including the previously poorly understood orphan NRs.

The NR4A family members are NR4A1, NR4A2 and NR4A3. They have various cellular functions and also act as molecular sensors. Depending on the cellular microenvironment, they can promote or inhibit cell death. All three receptors can bind to the same genomic *cis* elements. Knockout mouse models have been used to distinguish their inflammatory, metabolic and neurological functions, and their relationships to diseases.

Autophagy is a necessary, evolutionarily conserved process that helps maintain cell homeostasis by degrading unnecessary proteins and organelles. Its degradation of cytoplasmic substances is also essential in physiological development and the prevention of pathological conditions. Its dysfunction can lead to pathologies such as cancer and neurodegenerative diseases.

The NR4A subfamily is widely involved in disease-associated cell autophagy. The three subfamily members are effective regulators of glucose, glycogen utilization and gluconeogenesis, thus contributing to glucose homeostasis. They are also involved in controlling energy use in adipose tissue and may affect fat gain and body fat mass.

It has also been shown that NR4A subfamily members play roles in the onset of inflammation, as they are highly expressed in inflamed cells and tissues. They are also highly expressed in circulating monocytes and macrophages, including endothelial cells located at the site of atherosclerotic lesions, where they stimulate the formation of arterial plaques and the onset of atherosclerosis, increasing the chances of heart attack.

In addition, experimental data strongly suggest a correlation between the expression of the NR4A subfamily members and the onset of cutaneous melanoma, prostate cancer, breast cancer and uterine fibroids.

Nur77-mediated autophagy is mainly affected by the translocation of Nur77 from the nucleus to the cytosol or mitochondria, with mitochondrial localization having the strongest impact. Although no endogenous ligands of the NR4A subfamily members have been identified, several recent studies have revealed some compounds that can activate or inactivate Nur77 or induce nuclear export by binding to Nur77. The compounds triptolide, Csn-B and THPN have been shown to participate in Nur77-related autophagy. Compounds such as hydroxychloroquine, cilostazol and CYSC as well as heat stress can mediate Nurr1-related autophagy. In addition, Nor1 is widely involved in stress-related autophagy.

Understanding how autophagy is regulated by members of the NR4A family will reveal whether their dysregulation is related to human diseases. Novel drugs targeting the expression level, activity and nuclear export of NR4A receptors have therapeutic potential.

In summary, the three members of the NR4A subfamily regulate many biological processes and are potentially important pharmacological targets. In-depth research may help in the diagnosis, treatment and prognosis of NR4A family-related diseases.

## Data Availability

Further information on the literature search methods and databases used during this study are available from the corresponding author upon reasonable request.

## Abbreviations

AF1: Activation function-1
AR: Androgen receptor
ATG5: Autophagy protein 5
ATG12: Autophagy protein 12
C-DIM: 1,1-Bis(3′-indolyl)-1-(*p*-substituted phenyl) methane
CMA: Chaperone-mediated autophagy
COPD: Chronic obstructive pulmonary disease
Csn-B: Cytosporone B
CYSC: Cystatin C
DAX1: Dosage sensitive sex reversal adrenal hypoplasia congenital critical region on the X-chromosome, gene-1
DBD: DNA-binding domain
DDA: Dendrogenin A
GSK-3β: Glycogen synthase kinase 3 beta
hOSCP1: Human organic solute carrier protein 1
IL-1β: Interleukin-1β
Lamp-2A: Lysosomal-associated membrane protein2A
LBD: Ligand-binding domain
LXR: Liver X receptor
MAPK: Mitogen-activated protein kinase
MPTP: 1-Methyl-4-phenyl-1,2,3,6-tetrahydro pyridine
NF-κB: Nuclear factor kappa-B
Nor1: Neuron-derived orphan receptor1
NR: Nuclear receptor
NR4A: Nuclear receptor 4A
Nurr1: Nur-related protein 1
6-OHDA: 6-Hydroxydopamine
PPAR: Peroxisome proliferator-activated receptors
SHP: Short heterodimer partner
THPN: 1-(3,4,5-Trihydroxyphenyl)nonan-1-one
TNF-α: Tumor necrosis factor alpha
TRAF2: TNF receptor-associated factor2
VEGF: Vascular endothelial growth factor
